# The Prophylactic Value of Typically Normal Occlusion

**Published:** 1913-09-15

**Authors:** N. S. Hoff


### THE PROPHYLACTIC VALUE OF TYPICALLY NORMAL OCCLUSION.

BY N. S. HOFF, D.D.S.

EFFICIENCY OF TYPICAL OCCLUSION. It would seem almost axiomatic to say that efficiency in a mere machine, such for instance as a tread mill, or a more complicated machine like a watch, was altogether in proportion to the perfection of adaptation of its parts. A watch with one of its wheels in malocclusion, or with even one of the cogs of its most insignificant wheel defective, would not inspire confidence in its ability to determine train time, for occasionally it is quite important that we know the exact time we should be at the railway station. Is it not a fact that most owners of watches take special care and spend much money to possess and maintain in perfect working condition a mere machine for marking time, which necessarily has no considerable vital relation or function, and at the same time their dental apparatus on which health and happiness if not length of life, actually depends is tolerated with supreme confidence when it develops in a typical form and therefore executes its function indifferently or in some cases most ineffectively. It is the purpose of this discussion to demonstrate if possible that a normal and typical development of the teeth and jaws provide the most logical and acceptable scheme of oral prophylaxis, and that we shall obtain preventive dentistry more satisfactorily by cooperating with the physiological methods rather than by depending so largely on the technical resources of our present endeavors.

We have in previous papers called attention briefly to the embryonic development of the teeth and associated organs, and incidentally referred to such artificial interference as could be employed to secure better typical development, and we now wish to set forth as clearly as may be practicable the influence of the highest type of development on tooth health as well as the general systemic welfare. We believe typical articulation and normal occlusion will best secure the desired results.

The typical jaw for the human has probably been as well established by the studies of Bonwill and others as may be necessary for our purpose, and the application of his findings to the correction of malocclusion by Hawley* will be sufficient to give us accurate ideals and suggest proper technical method of attaining them.

From the dietetic view point it is essential that the mastication function shall be as perfect as is possible, for the reason that a proper food imperfectly masticated and insalivated will be imperfectly digested and assimilated. We must therefore maintain the dental arches intact so far as the normal complement of teeth is concerned; and the teeth should be in typical alignment so that the excursions of the mandible as influenced by the muscle action shall harmoniously bring the masticating surfaces of the teeth into effective contact relations without nervous shock or mental consciousness and especial effort by the patient. Mere articular relations of the teeth in symetrical arches does not mean efficiency in the masticating function, there must be harmony with muscular and nervous reaction, that automatically and somewhat unconsciously the whole function of mastication and insalivation with deglutition may be an agreeable and complete function. It should not be accomplished with conscious determination or fatigue. All this is only possible when the teeth are typically in normal occlusal and anatomical relations.

Because of their characteristic elasticity the muscles are to some extent adaptable as are most of the other involved organs, and it is remarkable that in some cases great aberrations in the position of the teeth in the jaws and even abnormally developed jaws will be over-

---

*See Transaction of Fourth International Dental Congress, Vol. II, page 252, and *Dental Cosmos*, May 1905. Also for statement of typical occlusion, Angle's Work on Malocclusion, Chapter I.

come by a process of accomodation on the part of the more adaptable organs. There will sometimes occur resorption and extra growth of the bone tissue to overcome malposition of the teeth, that the function of mastication may be at least partially accomplished. If such cases however are closely observed it will be found that the constant effort to accomplish this unnatural function has produced pathologic conditions of other structures, such as a lack of development to the normal type many times associated with impaired nerve function. Impaction of the third molars with a long train of local and reflex malnutritional influences, and serious referred pains to other more sensitive organs and tissues are more frequent results of abnormal dental relation than have been generally credited.

It may seem at first that it would be impracticable to undertake to determine the type of the jaw, or define the form of the dental arch, at the age of from six to twelve years with the idea of assisting by artificial means the development of the ideal jaw or arch. This might be a questionable procedure for the novice or superficial observer; but there is no reason why even at this stage of development we may not form a tolerably correct estimate of the tempermental type of the individual from his general physical development, a composite of his parents, and especially from the form and surface markings of the matured teeth that have already erupted. There certainly would be little chance for serious error when there is evidently a pronounced malformation of the jaws and malocclusion of the teeth. If there is a question of doubt nothing more need be done than to adjust at the earliest possible tine the erupting teeth into a symmetrical arch with approximating if not absolute occlusion. This will make it much easier for nature to bring about a typical alignment and a correct and at least an effective occlusion. It will also be found that much of the nervous strain through which children pass during this time will be lessened, and in most cases they pass it without serious notice or bad results. It is a common notion that children should not be subjected to orthodontic treatment during this critical period, but it is the testimony of all orthodontists that when the treatment is properly carried forward that no injury results, but on the contrary a large majority of the patients escape many customary nervous disorders peculiar to this period and they are prepared to start the adolescent period with the best possible chance for completing it with a most satisfactory maturity.

The contention we urge is that as soon as the permanent teeth begin to make their appearance at six or seven years constant professional supervision is necessary and appropriate orthodontic interference should be instituted as soon as it is indicated, to the end that at twelve to fourteen years of age all the teeth developed at that period should be in as nearly normal and typical alignment and occlusion as can be determined at that time. The most important consideration being that the arches will be large enough to prevent impaction or torsal relations, and that while the supporting osseous tissues are in the most active formative period there will be the best opportunity for typical alignment and adjustment to normal occlusion; at the same time the alveolar process will be developed to its normal condition to best support the teeth when they are brought into active service. If the orthodontic interference is applied early and not excessively no considerable pain need be produced and better bone development will result from the gentle stimulation of the force applied making less after retention necessary. It the teeth are properly aligned as they erupt they will be more likely to occlude normally and the function of mastication will be assured to the benefit of digestion and general food assimilation and tissue building throughout the body as well as in the teeth and jaws.

The value of typically developed jaws and teeth in normal relations has not been appreciated either by the profession or our patients. It is probably safe to say that 90 per cent of the cases of malocclusion treated by orthodontists come to them because of the facial deformities which are so displeasing to the parents of the children, and it may also be said with equal safety that the general practitioner does take little notice and makes no mention of what he too often considers such slight aberrations that they will never be noticed or as he says are not likely to be in any way injurious. There are of course in many cases such slight errors that they never prove to be seriously harmful, but a slight torsal relation in an erupting tooth may be in the completed arch the cause of serious malocclusion. In many cases the harm of a single rotated tooth in one arch will not appear until caries or peridentitis appears. Then it develops that the cause of the caries or peridentitis can be traced to the slight shifting of the teeth so as to throw out of perfect adjustment enough teeth in both arches to cause serious injury from caries or peridental diseases, therefore it can be shown that the correction of malocclusion in the developmental period is important not alone for esthetic or dietetic reasons alone for serious systemic as well as local pathologic conditions can be traced directly to malformations of the jaws and dental arches. It is practicable to cause the permanent teeth to erupt into sufficient arches of at least proximate typical form, and it is also possible to adjust the occlusal relations so that they may become functionally harmonious. If this be done at the period of eruption of the teeth, preventive treatment of the best kind will result from the view point of esthetics as well as local and systemic health. Harmony of function, with the highest type of efficiency ought to produce the best physical and esthetic proportions in the face and at the same time be a most important factor in a like development of all other tissues and organs of the body; because there is no more important or pleasurable experience than comes from the ability to thoroughly masticate and assimilate palatable food. Food that is masticated with comfort and efficiency will be properly assimilated and built into the body tissue in right proportions.

Much has been written about normal occlusion as a prophylactic against dental caries and peridental affections, but it is doubtful if the profession and the general public will soon practically apply the principles involved as prophylaxis measures. A perfect set of teeth in typical form and occlusal relations is so rare that we get accustomed to abnormal conditions and pay only a passing attention to slight irregularities. A writer in one of the prominent dental journals describes at length a complete denture in the mouth of a young man saying that it was the only complete denture he had encountered in a practice of thirty years. If the normal is so rare as that it would seem an idle task for us to set our ideals on the attainment of that which we can not hope to realize in any greater number of cases. There is however no good reason why we may not in this day of intelligence and skillful attainment assist nature in the production of the ideal in far greater proportion than has been seen in the past. In fact the modern practice of orthodontia is accomplishing the ideal in a most satisfactory manner and is demonstrating the practicability of ideal occlusion in the human mouth.

We have the fact often cited that the teeth of animals do not decay because their teeth are in normal relation and are used as they were intended to be to masticate hard foods. The fact is that, the dog for instance, while not subject to caries in any considerable degree almost invariably becomes infected with peridental diseases to the extent of complete loss of his teeth. The probable reason for the lack of caries in the dog is that because of the conical shape of his teeth, the constant cleansing of their surfaces by the food excursions during mastication of tough foods prevents the adhesive glutinous materials which serve to organize microbic plaques which are the forerunners of all tooth caries; and the reason for the peridental inflammation is to be found in the fact that because of their conically shaped teeth the food excursions are always forced against the gums, sometimes with tremendous force, and so the gum attachment is either torn away or forced apically and any infectious matter that may be present will be strongly injected into the gum tissue and a wound produced, infection quickly following. Were it not for the sterilizing influence of the dogs saliva there is little doubt that dogs and other animals as well would be great sufferers from diseased gums. In the herbivorous animals the gum tissue is exceedingly thick and resistent to the hard foods used by such animals. In some measure the dog's gum tissue is either naturally so formed or it becomes quite tough and resistent depending on the kind of food he is given to eat.

In man we have a tooth shaped so that the gums are in a sense protected from food contacts; like the pig we are omniverous and our teeth are fortunately well adapted to the mastication of a large variety of food materials. On the contrary the gums not being exposed to the hard usages of other animals are less thick and not so closely organized. In the molar region where the bulk of mastication is accomplished the cervical part of the crowns are constricted while the morsal surfaces are expanded to an inverted cone form. This provides a large grinding surface with the proximal borders in close contact leaving larger cone-shaped interproximal spaces into which food forced through the interproximal margins passes to be washed away by the saliva. It will thus readily be seen that food forced into these interproximal spaces by the masticating process will be easily and certainly eliminated providing the spaces are normal in size and form and the gum festoon has not degenerated from injury or disease. It is coming to be a recognized fact that peridental infections are frequently caused by injuries resulting from lack of proper contact of the proximal surfaces of the grinding teeth, either from proximal caries and imperfect restoration by filling, or because of loss of a tooth, or the malposition of one or more teeth. So important is this marginal contact that it has been suggested that the fillings of amalgam or cast gold shall be carved to imitate as nearly as possible the normal marginal ridge.*

Another common source of peridental irritation and in-

---

*See article by R. W. Bunting, Dental Summary 1912, page 878, and article by J. L. Young, Items of Interest, May 1913.

fection is from the deposits of calculus and mucoid substance at the servical portions of the crown, especially where those deposits are not washed or brushed away by the saliva, the tooth toilet or the food excursions. These deposits occur in the interproximal embrasures especially of malposed teeth, or teeth which have been badly filled, or teeth in which the fillings have disintegrated leaving pockets or rough surfaces which prevent self cleansing and provide conditions favorable for extraneous deposits. Gum infection and disease is inevitable under such conditions, whereas if the teeth were in proper alignment and free from caries or imperfect fillings the condition would not be favorable for deposits, or injury from food forced onto the unprotected gums during mastication. If these deposits of either character are excessive they increase in quantity and incroach upon the gingival margins producing mechanical irritation and the gums recede or become infected from the microorganisms contained in the deposits. If the teeth were in proper alignment and of proper form, with accurate contacts there would be less tendency for the accumulation of these inimical deposits and the natural cleansing by food and saliva would eliminate beginning deposits before they had a chance to cause peridental injury by impaction and infection.

PREVENTION OF CARIES. From the re-searches of Professor Miller and others we have fairly well established the method of carious infection, but the problem of its inhibition or prevention is still unsettled, but is being actively studied. The fact that tooth caries always has a local causal factor in defective enamel from injury or developmental faults, or because of abnormal relations of the proximating surfaces of the crowns is so well established that it would seem unnecessary to restate the matter here. But from the prophylaxis view point we find that it is highly important that these local factors be thoroughly emphasized, because preventive treatment is largely directed to the elimination of these predisposing causes. If the teeth were in normal alignment and without flaws of any sort there would be slight occasion for technical interference of the professional kind. Incipient caries is found generally in the fissures of the morsal surfaces of the masticating teeth, then at the proximal contact points, and then on the buccal surfaces near the gingival margins. The reasons for this seems to be that the fissues, because of incomplete calcification of the enamel at those points and the difficulty of preventing deposits of mucoid substances which become infected, supplying every facility for microbic proliferation, and because of the contracted fissures little chance for the saliva to neutralize the acids produced by the fermenting processes, this situation furnishes ideal conditions for the propagation of caries. A similar situation is found just below the proximal contact points of all the teeth. It is not at the actual contact point that caries begin, but at the point where the proximating surfaces of the teeth begin to diverge to form the cone-shaped interdental space. Here is provided another narrow fissure which is not kept free from deposits by the rubbing together of the tooth surfaces as at the actual contact point. This position is also protected from the food incursions which pass through the embrasures, and it is not reached by the tooth brush or saliva. Here the glutinoid deposits rest secure until micro-organisms begin to develop in the protecting inclosure of the gelatinous plaques. The acid generated by the fermentation process are held in close contact with the tooth surfaces and soon etch their way into the tooth substance until a cavity is formed which securely protects and encourages further disintegration of the entire tooth. The same method is followed where because of malposition of one or more teeth a favorable situation is provided for the beginning of the process of disintegration. Similar conditions are found on the buccal and labial surfaces of the teeth near the gum line, although caries in not so prevalent in these situations, because there is greater opportunity for the lips, cheek and tooth brush to keep these positions free from the inimical deposits.

Obviously the treatment for such conditions in the professional correction of conditions that favor the plaque and glutinous deposits. This is done by eradicating the fissures by grinding them out so smooth that food and other deposits will be kept out of them by the masticating process or if too deep by filling them and making them self cleansing. The proximal crypts should be dressed smooth and highly polished and the patient instructed to polish them thoroughly at least once each week with floss silk saturated with a suitable but fine abrasive tooth powder. The labial and buccal points of attack should be polished carefully by the dentist and the patient instructed as to the proper use of a polishing stick which should be used at sufficiently frequent periods to prevent deposits getting an attachment. At times it may be best to touch such places, particularly on the inaccessible buccal surfaces of the third molars, with nitrate of silver to secure disinfection and the formation of a protecting eschar.

Various expedients can be utilized to overcome such developmental conditions as are liable to be present in dentures that seem to be perfectly formed and are in correct automatic relations. We shall give more in detail the individual and professional prophylaxis method in another paper.

